# Hydroxyurea Use and Hospitalization Trends in a Comprehensive Pediatric Sickle Cell Program

**DOI:** 10.1371/journal.pone.0072077

**Published:** 2013-08-14

**Authors:** Kerri A. Nottage, Jane S. Hankins, Matthew Smeltzer, Fawaz Mzayek, Winfred C. Wang, Banu Aygun, James G. Gurney

**Affiliations:** 1 Department of Hematology, St. Jude Children's Research Hospital, Memphis, Tennessee, United States of America; 2 Department of Biostatistics, St. Jude Children's Research Hospital, Memphis, Tennessee, United States of America; 3 Division of Epidemiology and Biostatistics, School of Public Health, University of Memphis, Memphis, Tennessee, United States of America; 4 Division of Pediatric Hematology/Oncology and Stem Cell Transplantation, Steven and Alexandra Cohen Children's Medical Center of New York, New Hyde Park, New York, United States of America; 5 Department of Epidemiology and Cancer Control, St. Jude Children's Research Hospital, Memphis, Tennessee, United States of America; Southern Illinois University School of Medicine, United States of America

## Abstract

**Background:**

A decline in hospitalizations and pain episodes among those with sickle cell disease (SCD) who take hydroxyurea (HU) has been shown when compared to pre-HU patterns but paradoxically, when compared to those who have never been treated, HU recipients often have more frequent hospitalizations. This analysis evaluates the impact of increasing usage of HU on trends in hospitalizations and blood transfusions within a large SCD treatment program.

**Methods:**

Eligibility was restricted to patients with Hb SS or Hb Sβ^0^-thalassemia who were 2–18 years old between 2006–2010 and received care at St. Jude Children's Research Hospital (N = 508). Hospitalizations and blood transfusions were calculated for each of the years under study for those exposed and never exposed to HU. Differences in number of hospitalizations before and after HU initiation were compared.

**Results:**

The proportion of patients receiving HU increased by 4% per year on average. In the HU exposed group, a modest decline in mean per-patient hospitalizations and per-patient hospital days occurred, while those never exposed to HU trended toward a slight increase over time. Rates of blood transfusions declined among those on HU but not in patients never exposed to HU. Patients on HU had a median of one fewer hospital admission in the year after initiation of HU, compared to the year prior. Two deaths occurred in the patient population, both of whom were not exposed to HU.

**Conclusions:**

Increasing usage of HU was concurrent with decreased hospitalization rates and blood transfusions. Our results support the utility of HU on decreasing hospitalizations and transfusions for patients with SCD outside of the clinical trial setting.

## Introduction

The clinical benefits of hydroxyurea (HU) for decreasing sickle cell disease (SCD)- related complications are well documented, and include a reduction in vaso-occlusive pain events, acute chest episodes, and mortality.[1]–[3] Clinical trials have shown a decrease in hospitalizations among patients with SCD on HU, although evaluations of health care usage outside of the clinical trial setting have been conflicting. A decline in hospitalizations and pain episodes among those who take HU has been shown when compared to pre-HU patterns but paradoxically, when compared to those who have never been treated, HU recipients often have more frequent hospitalizations.[4], [5] These findings are limited by short duration of follow-up and focused selection of severe clinical phenotypes. HU has been shown to be underutilized by care providers.[5], [6] Data to support the effectiveness of HU in conserving costly health care resources are valuable to justify increased usage of HU. To minimize common limitations of prior studies, the effect of HU on inpatient resources in the SCD population may be investigated by examining the trend in health care utilization when there is variability in the proportion of hydroxyurea users over time.

Accordingly, this analysis evaluated trends in health care utilization rates (hospitalizations and blood transfusions for acute complications) and mortality among patients with SCD at St. Jude Children's Research Hospital (St. Jude). At St. Jude, HU is prescribed for routine clinical care using consensus guidelines[7] which minimizes variability in prescribing by the clinicians. We hypothesized that hospitalization rates would decrease as use of HU increased among our pediatric SCD population.

## Methods

Since 1997 a database has been maintained at St. Jude to collect SCD-related outcome data. Variables captured in the database include patient demographics, sickle cell genotype, hospitalizations, and date and cause of death. Also collected are data regarding sickle cell therapies, such as blood transfusions (both intermittent and chronic), hematopoietic stem cell transplant, and HU therapy. Fewer than ten percent of hospitalizations occur outside our treating institutions; information about these events was obtained initially by patient/family self-report, then validated through documentation from the treating institution. Permission for analysis of these clinical data for research purposes was granted by the St. Jude Institutional Review Board with a waiver of consent from participants.

Patients included in this analysis were between the ages of 2 and 18 years with Hb SS or Sβ^0^-thalassemia genotypes and were treated at St. Jude between the years 2006–2010. Discharge diagnoses that were included in this analysis were pain/vaso-occlusive episodes, acute chest syndrome, stroke, splenic sequestration, priapism, aplastic crisis, and sepsis. The St. Jude HU treatment consensus guidelines were followed to guide selection of patients for treatment. The indications outlined in the consensus guidelines are for patients with Hb SS and Hb Sβ^0^-thalassemia who are age ≥24 months and have any of the following - pain events including dactylitis, acute chest syndrome, low Hb F percentage, elevated WBC count or LDH, chronic hypoxemia, or abnormal transcranial doppler and refusal of chronic transfusion therapy. Other indications are discussed on a case-by-case basis and include conditional transcranial dopplers, abnormal MRI suggesting silent infarcts, neurocognitive decline, poor growth and development, age <24 months, Hb SC disease, and parental request. Those never prescribed HU did not meet any of the above criteria or declined the therapy.[7]

The number of patients who satisfied the inclusion criteria on July 1^st^ of each year during the 2006–2010 period was calculated. Information abstracted included number of hospitalizations, packed red blood cell (PRBC) transfusions, and deaths. Patients on chronic transfusion therapy were included in this analysis; however only transfusions given for an acute complication were calculated. Patients were classified in two subgroups: those who received hydroxyurea at any point during the period of the analysis (exposed group), and those who never received this treatment (never exposed group). Patients who received HU were included in the exposed group only from the year of initiation onward, and were not included in the never exposed group at any time point.

### Statistical Analysis

**Figure 1:**
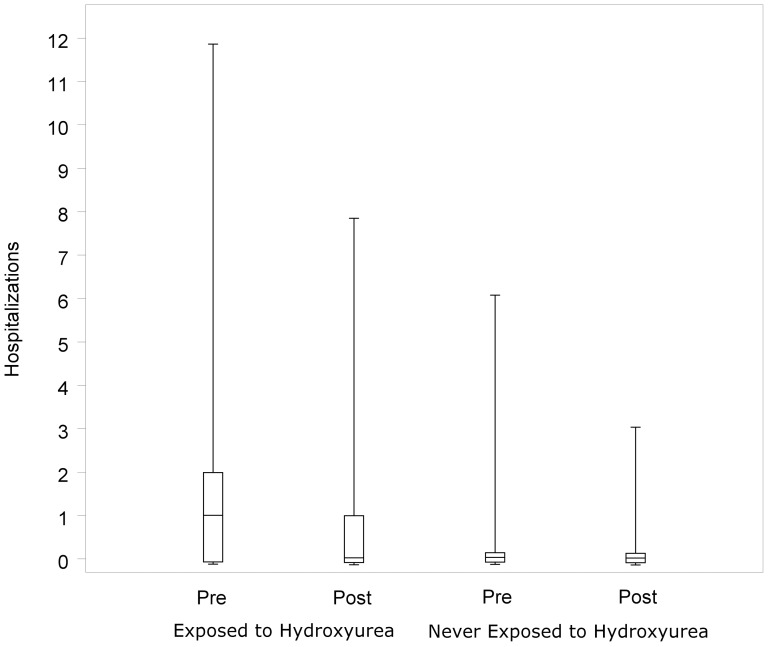
Boxplots show the distribution of hospitalizations by hydroxyurea exposure status for the time period pre- and post- initiation of HU or pre- and post- the study mid-point. Subjects exposed to HU (n = 205) experienced a median decrease of 1 hospitalization per year after initiation of HU (p<0.001). Subjects never exposed to HU (n = 197_ experienced no discernible change in yearly hospitalizations (p = 0.30) for the years immediately preceding and following the study mid-point. Boxplots show the minimum, 1^st^ quartile, median, 3^rd^ quartile, and maximum for each group after a jittering technique was applied to aid the visual presentation of overlapping data points.

The number of occurrences of each clinical variable of interest was calculated for each year. Annual rates of events are presented as overall number per year and total numbers of subjects with a specific event per year. Differences in the median number of hospitalizations one year before and one year after starting HU therapy were compared using the Exact Wilcoxon Signed Rank test. For the never exposed group the median number of hospitalizations was compared one year prior to January 1, 2008 (the approximate study mid-point), and one year after. Boxplots in Figure 1 show the minimum, 1st quartile, median, 3rd quartile, and maximum for each group after a jittering technique was applied to aid the visual presentation of overlapping data points.[8]

## Results

Inclusion criteria were met for 508 patients, with 94% having Hb SS and 6% Hb Sβ^0^-thalassemia (Table 1). Males comprised 53% of the study population. The median age was 10 years (range 2–18.5) at the midpoint of the study period. The trend in HU use during the study period is also shown in Table 1. The proportion of patients who received HU increased by 4% per year on average, with 28% of patients prescribed HU in 2006 and 43% in 2010.

**Table 1:** Characteristics of the pediatric SCD population eligible for treatment with hydroxyurea from 2006–2010.

	Total Population N = 508	Exposed to Hydroxyurea N = 203	Never Exposed to Hydroxyurea N = 305
**Sex**			
Female	241 (47%)	86 (42%)	155 (51%)
Male	267 (53%)	117 (58%)	150 (49%)
**Age** * **(years)**			
Median (range)	10.0 (2.0, 18.5)	10.8 (2.1, 18.5)	9.6 (2.0, 18.4)
**SCD genotype**			
SS	479 (94%)	192 (95%)	287 (94%)
Sβ^0^-thalassemia	29 (6%)	11 (5%)	18 (6%)
**Year of birth**			
1990–1994	121 (24%)	56 (28%)	65 (21%)
1995–1999	135 (27%)	75 (37%)	60 (20%)
2000–2004	129 (25%)	52 (26%)	77 (25%)
2005–2009	123 (24%)	20 (10%)	103 (34%)
**Interval year of analysis**	**Proportion on hydroxyurea**		
2006	28%		
2007	32%		
2008	37%		
2009	41%		
2010	43%		

*Age at study midpoint (2008).

Among those in the HU-exposed group, there was a progressive decrease in the average per-patient hospitalizations per year and average number of hospitalizations among those who were hospitalized. (Table 2) In contrast, the per-patient hospitalization rate among the patients not exposed to HU remained stable, and the hospitalizations among those who were hospitalized trended toward a slight increase over time. Average per-patient hospital days declined in both groups, but the decline was greater in the HU exposed group. Patients exposed to HU had more hospitalizations and more days in the hospital per patient than the unexposed group. With regard to PRBC transfusions, patients exposed to HU received 1 fewer transfusion per year at the end of the observation period (2010), than they did in the first year (2006), while the number of transfusions received by each patient in the never exposed group remained unchanged. (Table 3) There were only two deaths that occurred over the study period and both occurred in patients never exposed to HU. Both deaths occurred after cardiopulmonary arrest, one in the setting of influenza H1N1, and the other due to severe cardiomyopathy secondary to transfusion-related iron overload.

**Table 2:** Hydroxyurea usage and hospitalizations.

	Ever Exposed to Hydroxyurea				Never Exposed to Hydroxyurea			
Year	N	Hospitalizations per patient per year	Hospitalizations per hospitalized patient	Hospital days per patient per year	N	Hospitalizations per patient per year	Hospitalizations per hospitalized patient	Hospital days per patient per year
2006	105	1.0	2.4	3.4	197	0.3	1.2	1.0
2007	127	1.0	2.2	3.4	207	0.3	1.3	1.1
2008	156	0.8	2.3	2.8	229	0.3	1.4	1.0
2009	178	0.7	1.9	2.8	241	0.3	1.4	0.9
2010	189	0.6	2.2	2.8	255	0.3	1.5	0.8

**Table 3:** PRBC* transfusions among those never exposed, and those exposed to hydroxyurea.

	Ever Exposed to Hydroxyurea		Never Exposed to Hydroxyurea	
Year	Proportion of Patients Transfused	Transfusions/Transfused Patients	Proportion of Patients Transfused	Transfusions/Transfused Patients
2006	4%	2.0	12%	1.5
2007	4%	3.4	9%	1.4
2008	3%	1.8	10%	2.0
2009	2%	1.0	8%	1.4
2010	2%	1.0	10%	1.5

*PRBC =  Packed Red Blood Cells.

To directly assess the impact of HU on the number of hospitalizations, an analysis was conducted on a subset whose hospitalization data for a full year prior to initiation of HU therapy and a full year after drug initiation was contained within the 2006–2010 date range. (n = 172). There was a statistically significant difference in the median number of hospitalizations in the year pre- and post-initiation of HU with 1 fewer admission to the hospital after starting therapy (p<0.001; Figure 1). In the never exposed group (n = 197), a similar analysis was performed using the year prior to the study mid-point and the year thereafter and no discernible change in median number of hospitalizations was observed (p = 0.30).

## Discussion

Our results show an increasing proportion of patients using HU among this pediatric SCD population between 2006 and 2010; a concurrent downward trend in the rates of inpatient hospitalizations over the same time period was observed. In the HU exposed group, there was a modest decline in per-patient hospitalizations and per-patient hospital days over the study period. In contrast, in those never exposed to HU the average number of hospitalization did not change over the time period, and number of hospital days per hospitalization appeared to slightly increase. Frequent hospitalizations and blood transfusions are burdensome and costly to insurance agencies and for families of patients with SCD.[9], [10] Our results further support HU use as a means of reducing these events and the resulting expense incurred.

The existing literature contains contradictory results related to the effectiveness of hydroxyurea therapy to improve hospitalization rates and health care utilization outside of the clinical trial setting. A study using a billing database in the state of Maryland showed that in the 5 years subsequent to FDA approval of HU for SCD there were significantly more hospitalizations of adult SCD patients when compared to 4 years prior to its approval. Investigators also showed that during the study period the cost of inpatient care for patients with SCD, regardless of HU use, increased almost 60% above inflation.[5] Another analysis using Medicaid data showed that patients on HU were admitted to a hospital more frequently than non-HU users (5 vs. 1.5 admissions per year, p = .004). However, patients who had the highest number of refills of HU had fewer hospitalizations than patients who rarely refilled their medicine (2.44 vs. 7.57 admissions, p = .043),[11] suggesting that hospitalizations are less frequent with more consistent use of the drug.

Health care utilization among pediatric populations, in contrast to adults, is not well characterized. Consistent with our results, data from a pediatric SCD cohort in South Carolina showed that individuals with SCD treated with HU had a reduction in hospitalizations compared to the time prior to HU initiation. However, when compared to those not treated with HU, patients on therapy had more frequent pain episodes, acute chest episodes, emergency room visits, and hospitalizations.[4] These inconsistencies likely indicate an imbalance in disease severity between the groups, which makes fair comparisons a challenge.

There are several strengths of this analysis. Management of SCD with HU is subject to variability among different institutions and providers so this large cohort of patients with unvarying hospital policies/procedures and a uniform approach to HU therapy provides consistency that may not be present in multi-institutional studies. In addition, these patients were all treated outside of the context of an interventional clinical trial of HU, so our findings represent a practical, “real world” application of the use of this therapy.

Additionally, the existing St. Jude HU consensus guidelines[7] allow for consistency of prescribing practices among our clinicians which, in most other settings, tends to be influenced by variable beliefs surrounding HU and unstandardized definitions of disease severity warranting treatment.[6], [12] For example, a survey of pediatric hematology/oncology providers identified through the American Society of Hematology/Oncology demonstrated that 42% of prescribers only consider painful crises that require hospitalization when deciding on a patient's eligibility for the drug, leaving pain managed at home unaccounted for.[13] In another survey of adult hematology/oncology providers in active practice, 36% reported they doubted the effectiveness of HU.[12]

Our results should be interpreted with awareness of some limitations. The investigation was completed as a secondary analysis of existing data that was collected for non-research purposes. Patient adherence to HU therapy could not be accounted for in the analysis, so it is not known the degree to which compliant patients fared better than those with poorer compliance rates. Also, some patients may have been taken off HU due to toxicity or poor adherence with clinic visits during the study period and this time off the drug could not be accounted for either. Suboptimal drug adherence or early discontinuation of therapy would cause hospitalizations to be overestimated among those on HU. Furthermore, the difference in hospitalization rate pre- and post-HU initiation was likely underestimated because patients generally do not achieve full clinical benefit from HU until they reach maximum tolerated dose (MTD), which can take six months to a year.[2], [14], [15]

Another limitation is that the available data did not capture all SCD-related complications that occurred outside of the hospital or in emergency rooms. Characterizing an imbalance in severity phenotype between those ever exposed to HU and those never exposed is incomplete without this information, though the imbalance expectedly exists. This inequity makes a direct statistical comparison between groups impractical and is the rationale for examining trends between the two groups as an alternative analytic approach.

Despite ample evidence in the clinical trial setting that HU is effective and well-tolerated, treatment has been underutilized in patient populations.[6], [12], [13], [16] This underutilization limits the ability to fully realize the effectiveness of HU in everyday clinical practice. Our results support the utility of HU on decreasing hospitalizations and transfusions for patients with SCD outside of the clinical trial setting. Fewer hospitalizations allows for conservation of inpatient resources and, although a cost analysis was not performed, it is reasonable to presume that decreasing hospitalizations and fewer blood transfusions for acute complications of SCD would result in an overall cost savings.[17]–[21] Moreover, with fewer hospitalizations, pediatric patients with SCD (and their parents) would, in turn, have fewer days of missed school, missed work, lost income, and lost productivity, and could more fully contribute to society.
